# No inhalation in combination with high frequency ventilation treatment in the treatment of neonatal severe respiratory failure

**DOI:** 10.12669/pjms.325.10682

**Published:** 2016

**Authors:** Xiaohui Guo, Yanfeng Sun, Jing Miao, Min Cui, Jiangbo Wang, Shuzhen Han

**Affiliations:** 1 Xiaohui Guo, Department of Pediatrics, Binzhou People’s Hospital, Shandong 256610, China; 2 Yanfeng Sun, Department of Oncology, Binzhou People’s Hospital, Shandong 256610, China; 3 Jing Miao, Department of Pediatrics, Binzhou People’s Hospital, Shandong 256610, China; 4 Min Cui, Department of Pediatrics, Binzhou People’s Hospital, Shandong 256610, China; 5 Jiangbo Wang, Department of Pediatrics, Binzhou People’s Hospital, Shandong 256610, China; 6 Shuzhen Han, Department of Pediatrics, Binzhou People’s Hospital, Shandong 256610, China

**Keywords:** Newborn, Severe respiratory failure, iNO

## Abstract

**Objective:**

To discuss over NO inhalation (iNO) in combination with high frequency ventilation treatment in relieving clinical symptoms and respiratory state of patients with neonatal severe respiratory failure.

**Methods:**

Ninety newborns with severe respiratory failure who received treatment in our hospital were selected for this study. They were divided into research group and control group according to visiting time. Patients in the control group were given conventional treatment in combination with high-frequency oscillatory ventilation, while patients in the research group were given iNO for treatment additionally besides the treatment the same as the control group. Changes of respiratory function indexes and arterial blood gas indexes of patients in the two groups were compared. Mechanical ventilation time, time of oxygen therapy and the length of hospital stay were recorded. Besides, postoperative outcome and the incidence of complications were analyzed.

**Results:**

After treatment, the level of PaO_2_ of both groups significantly improved, and respiratory function indexes such as partial pressure of carbon dioxide in artery (PaCO_2_), oxygenation index (OI), fraction of inspiration O_2_ (FiO_2_) and mean arterial pressure (MAP) decreased (P<0.05); the improvement of various indexes of the research group was more obvious than that of the control group (P<0.05). Mechanical ventilation time, oxygen therapy time and the length of hospital stay of the research group was much shorter than those of the control group. The incidence of complications in the two groups had no statistically significant difference (P>0.05), but the clinical outcome of the research group was better than that of the control group.

**Conclusion:**

NO inhalation in combination with high frequency ventilation for treating neonatal severe respiratory failure is effective in improving blood gas index and respiratory function, enhance cure rate, and reduce the incidence of complications and mortality; hence it is safe and effective and worth clinical promotion.

## INTRODUCTION

Meconium inhalation syndrome, neonatal pneumonia, asphyxia and respiratory distress syndrome are the commonly seen diseases which can induce neonatal respiratory failure, which severely threatens health of children.^1^ Most newborns who are admitted into neonatal intensive care unit can have improved respiratory function and recovered oxygenation after conventional airway nursing and normal frequency mechanical ventilation; however, a few newborns with severe disease condition may have unrelieved clinical symptoms after undergoing the above treatment but have to turn to other intervention treatment to improve prognosis and lower death rate.^2^,^3^ Currently, treatment schemes for improving ventilation of newborns with respiratory failure include high-frequency oscillatory ventilation, the supplement of pulmonary surfactant, nitric oxide (NO) inhalation, liquid ventilation and extracorporeal membrane oxygenation.^4^ Those treatment strategies aim at gathering more pulmonary alveoli, inhibit pulmonary injury induced by high airway pressure, improve oxygenation state, increase pulmonary blood flow, reduce vessel shunt in lung, and increase ventilation/perfusion ratio.^5^,^6^ NO inhalation and high-frequency oscillatory ventilation have been attracted much attention in recent years for advantages of simple operation and low cost.

NO, a kind of vasoactive substance generated and released by vascular endothelial cell, has extensive biological activity. It can relax pulmonary artery smooth muscle and bronchi smooth muscle, lower pulmonary arterial pressure and airway pressure, relieve pulmonary inflammatory reaction induced by endotoxin, reduce the release of cell factors and mediators, and stop the occurrence and development of immune injury and inflammatory reaction.^6^,^7^ European countries and America have applied inhaled NO to treat pulmonary hypertension, pulmonary fibrosis, and persistent pulmonary hypertension of newborn, respiratory distress syndrome and acute hypoxemic respiratory failure.^8^,^9^ This study treated neonatal severe respiratory failure with NO inhalation in combination with high-frequency oscillatory ventilation and obtained good curative effect.

## METHODS

Ninety newborns with severe respiratory failure who were admitted into the Binzhou People’s Hospital from May 2012 to 2014 were randomly selected, including 41 cases of type I respiratory failure and 49 cases of type II respiratory failure. There were 58 premature infants and 33 full-term infants. All included newborns conformed to the diagnostic criteria of severe respiratory failure and did not recover after conventional respiratory supportive treatment. Parents of those newborns gave signed informed consent. Newborns with severe multiple malformation or severe congenital heart disease were excluded. The newborns were divided into research group and control group according to admission time. In the research group, there were 23 boys and 22 girls, with gestational age of 30 ~ 38 weeks (average 34.70±3.60 weeks), weighed 1610 ~ 3050 g (average 2334.50±720.90 g), and aged 1 ~ 24 h on admission; there were 25 cases of respiratory distress syndrome, 9 cases of meconium aspiration, 7 cases of pneumonia and septicemia, three cases of asphyxia, and one case of Wilson-Mikity. In the control group, there were 26 boys and 19 girls, with gestational age of 32 ~ 39 weeks (average 35.20±5.40 weeks), weighed 1623 ~ 2976 g (average 2411.30±812.60 g), and aged 1 ~ 24 h on admission; there were 23 cases of respiratory distress syndrome, 11 cases of meconium aspiration, 6 cases of pneumonia and septicemia, three cases of asphyxia, and two cases of Wilson-Mikity. Differences of general data between two groups had no statistical significance; hence, the results were comparable. This study followed the ethics management regulations of the Binzhou People’s Hospital.

### Therapeutic method of the control group

Patients in the control group were treated with conventional treatment and high-frequency oscillatory ventilation. High-frequency oscillatory ventilation was performed using Stephanie pediatric ventilator (Germany) in high-frequency oscillatory mode. Mean airway pressure (MAP) was set as 1 ~14 cm H_2_O, vibration pressure amplitude was set as 35 ~ 45 cm H_2_O, vibration frequency was set as 9 ~ 10 Hz, and inspiratory time was set as 33%. Pulmonary reexpansion was achieved by improving MAP in an amplitude of 1 ~ 2 cm H_2_O until pulmonary reexpansion reached the 8^th^ and 9^th^ posterior ribs.

### Therapeutic method of the research group

Besides the treatment the same as the control group, patients in the research group was also treated with NO inhalation treatment. The initial concentration of NO was 15 ppm; transcutaneous oxygen saturation (TcSaO_2_) equal to or higher than 90% was taken as the baseline. After 30 ~ 60 min of NO inhalation, if TcSaO_2_ increased for more than 10% and alveolar oxygen tension (PaO_2_) increased for more than 10 mm Hg (1 mm Hg=0.133 kPa), then the treatment was determined as effective; otherwise, the treatment was determined as ineffective. For patients who obtained effective treatment result, the concentration of NO was decreased for 2 ~ 3 ppm, while for those who obtained ineffective treatment result, the concentration of NO was increased for 2 ~ 3 ppm till TcSaO_2_ and PaO_2_ became stable. The concentrations of NO and FiO_2_ were reduced every 6 to 12 hour if disease conditions of patients improved. If the concentration of NO decreased to 3 ppm and moreover FiO_2_ stayed equal to or lower than 30% and TcSaO_2_ stayed equal to or higher than 90% for 12 h, then NO inhalation stopped.

### Evaluation standard

The outcome was evaluated referring to the standards mentioned in Pediatric Respiratory Failure Foundation and Clinic.^10^ If clinical symptoms and signs disappeared or basically disappeared, characteristic changes of chest X-ray film disappeared, vital signs became stable, and patients could breathe by themselves, then the disease could be determined as cured. If characteristic changes of chest X-ray film showed significant improved after treatment, vital signs tended to be stable and respiratory support strength decreased, then the disease condition was determined as relieved. If characteristic changes of chest X-ray films remained unchanged, clinical symptoms and signs had no changes or continued to deteriorate, and patients still needed the support of breathing machine, then treatment was determined as ineffective. If patients died after treatment, then the outcome was death.

### Observation index

Respiratory functional indexes such as oxygenation index (OI), Fraction of inspiration O_2_ (FiO_2_) and MAP and arterial blood gas indexes such as PaO_2_ and partial pressure of carbon dioxide in artery (PaCO_2_) were observed before treatment (T0), three hours after treatment (T1), 6 hours after treatment (T2), and 24 hours after treatment (T3). Mechanical ventilation time, oxygen therapy time and length of hospital stay of patients in the two groups were recorded. Postoperative outcome and the incidence of complications were analyzed.

### Statistical analysis

SPSS ver. 19.0 was used to analyze data. Measurement data were expressed as mean±SD. Comparison between groups was performed using t test. Enumeration data were expressed as percentage and processed by chi-square test. Difference was considered as statistically significant if P<0.05.

## RESULTS

### Comparison of arterial blood gas indexes of two groups

At T0, difference of arterial blood gas indexes had no statistical significance (P<0.05); at T1 to T3, the level of PaO_2_ of both groups improved compared to T0, but the level of PaCO_2_ decreased compared to T0, and the differences had statistical significance (P<0.05). The improvement of various indexes of the research group was more obvious than that of the control group, and the difference had statistical significance (P<0.05) (Table-I).

**Table-I:** Comparison of arterial blood gas indexes of two groups (mean±SD).

*Group*	*Time*	*PaO_2_ (mm Hg)*	*PaCO_2_ (mm Hg)*
Research group	T0	37.51±5.27	67.79±10.32
	T1	48.65±3.48*^#^	57.31±6.82*^#^
	T2	62.49±2.56*^#^	51.40±5.77*^#^
	T3	65.33±4.62*^#^	44.91±5.17*^#^
Control group	T0	37.53±5.31	67.81±10.29
	T1	43.32±3.38*	60.90 ±3.48*
	T2	56. 83±2.86*	55.36±4.72*
	T3	60.32±4.12*	49.34±5.12*

* *Note:* indicates P<0.05 compared to T0;

# indicates P<0.05 compared to control group.

### Comparison of respiratory function indexes of two groups

At T0, differences of respiratory function indexes between two groups had no statistical significance (P<0.05); at T1 ~ T3, respiratory function indexes such as OI, MAP and FiO_2_ of two groups decreased compared to T0; except for the difference of MAP before treatment and at T1, differences of the other indexes before treatment and at all-time points after treatment all had statistical significance (P<0.05); the improvement of various indexes of the research group was remarkable than that of the control group, and the difference had statistical significance (P<0.05) (Table-II).

**Table-II:** Comparison of respiratory function indexes of two groups (mean±SD).

*Group*	*Time*	*OI*	*FiO_2_ (%)*	*MAP (mm H_2_O)*
Research group	T0	33.49±8.92	88.59±15.78	14.69±0.96
	T1	14.61±3.84*^#^	73.66±10.62*^#^	14.06±0.90
	T2	12.96±3.34*^#^	62.96±7.52*^#^	13.81±0.92*^#^
	T3	10.62±3.04*^#^	51.64±6.93*^#^	13.41±1.20*^#^
Control group	T0	33.51±8.94	88.61±15.80	14.67±0.97
	T1	18.97±3.53*	80.65±10.37*	14.33±0.91
	T2	16.95±3.33*	72.66±7.35*	14.01±0.91*
	T3	13.19±2.92*	59.58±6.82*	13. 83±0.92*

*Note:* OI=PaO_2_/FiO_2_;

* indicates P<0.05 compared to T0;

# indicates P<0.05 compared to control group.

### Mechanical ventilation time, oxygen therapy time and length of hospital stay

Mechanical ventilation time, oxygen therapy time and length of hospital stay of newborns in the research group were shorter than those of newborns in the control group, and the differences had statistical significance (P<0.05) (Table-III).

**Table-III:** Comparison of mechanical ventilation time, oxygen therapy time and length of hospital stay between two groups (mean±SD).

*Group*	*Mechanical ventilation time (d)*	*Oxygen therapy time (d)*	*Length of hospital stay (d)*
Research group	4.54±1.48	9.36±3.24	28.34±4.33
Control group	6.63±1.97	13.49±3.27	37.72±4.41
t value	5.603	6.004	10.025
P value	<0.05	<0.05	<0.05

### Outcome and the incidence of complications

In the research group, there were two cases of pneumothorax, six cases of gastrointestinal hemorrhage/necrotizing enterocolitis, one case of pulmonary hemorrhage, 8 cases of multiple organ injury; the incidence of complications was 37.8%. In the control group, there were three cases of pneumothorax, five cases of gastrointestinal hemorrhage/necrotizing enterocolitis, two cases of pulmonary hemorrhage, and 10 cases of multiple organ injury (44.4%). The difference of the incidence of complications had no statistical significance (X^2^=1.627, P>0.05). As to postoperative outcome of the research group, 22 patients were cured, 17 patients relieved, three patients obtained ineffective treatment, and three patients died; in the control group, 14 patients cured, 20 patients relieved, six patients obtained ineffective treatment, and five patients died. The cure rate of the research group was higher than that of the control group, and the difference had statistical significance (X^2^=4.532, P<0.05).

## DISCUSSION

Neonatal respiratory failure is a commonly seen critical disease in neonatal intensive care unit and it is also an important cause of the death of newborns. It has been found that; respiratory distress syndrome has the highest incidence among newborns with gestational age equal to or larger than 34 weeks, followed by meconium aspiration syndrome, pneumonia/septicemia and wet lung disease.^11^ Previous multi-center epidemiological investigation results in China also suggest that, respiratory system disease, especially respiratory distress syndrome, is the most commonly cause for neonatal respiratory failure, and other causes include neonatal hypoxic ischemic encephalopathy, primary or secondary pulmonary hypertension and various congenital malformations.^3^ In the past twenty years, treating respiratory failure of critically ill newborns with conventional mechanical ventilation has gain great progress; however, some severe cases still cannot be relieved. The reason for failed treatment is that, the consumption of surface active material results in pulmonary collapse and the release of inflammatory mediators, barotrauma and shearing injury caused by mechanical ventilation aggregate lung injury, the release of endogenous NO of those patients is reduced, and moreover pulmonary arteriospasm can induce pulmonary hypertension; therefore, mechanical ventilation alone is lowly effective for some newborns with severe respiratory failure.^12^,^13^

NO inhalation treatment is a new kind of respiratory supportive means developed in recent years. NO, a kind of endogenous endothelium-derived relaxing factor has been found having a vital role in the pathological and physiological process of many diseases.^14^,^15^ Many clinical researches have found that, NO can be used for relieving ischemia reperfusion injury caused by lung transplantation, selectively lowering pulmonary artery resistance of patients undergoing heart transplantation and patients with congenital heart disease and treating severe hypoxic respiratory failure, high altitude pulmonary edema and acute respiratory distress syndrome.^16^ High frequency ventilation is based on low tidal volume and rapid frequency (10 ~15 Hz). Currently, the most effective high frequency ventilation is high-frequency oscillatory ventilation which can prevent alveolar collapse with stable MAP; to be specific, it can minimum the increase of airway pressure induced by tidal volume and the change of airway pressure is little because of the high working efficacy, which can protect the lung of newborn and avoid barotrauma.^17^ High frequency ventilation and NO inhalation can complement each other’s advantages and the combination of the two therapies can effectively improve ventilation-perfusion ratio.^18^

This study treats severe respiratory failure of newborns with NO inhalation and high frequency ventilation. Results suggested that, the improvement of respiratory failure associated clinical symptoms, respiratory function indexes and arterial blood gas indexes of the research group was superior to that of the control group which did not apply NO inhalation. It indicated that, high frequency oscillatory ventilation in combination with NO inhalation could effectively relieve clinical symptoms such as dyspnea, cyanosis and arrhythmia and it was positive in recovering OI and improving ventilation function. Besides, it was also found that, mechanical ventilation time, duration of oxygen therapy and the length of hospital stay of newborns who were treated with NO inhalation assisted respiratory support treatment was shorter than those of newborns in the control group, the outcome of the research group was better than that of the control group, and the incidence of complications of the research group and the control group had no statistical significance. The above findings suggested that, NO inhalation in combination with high frequency oscillatory ventilation had definite effect in treating severe respiratory failure of newborns and it could significantly shorten the duration of respiratory supportive treatment, without significantly increasing adverse reactions.

## CONCLUSION

Treating newborns suffering from respiratory failure with NO inhalation and high frequency oscillatory ventilation is safe and effective. It can effectively improve respiratory function and stage of newborns relieving related symptoms; therefore, the combined therapy is worth clinical promotion.
